# The soft X-ray monochromator at the SASE3 beamline of the European XFEL: from design to operation

**DOI:** 10.1107/S1600577522007627

**Published:** 2022-08-23

**Authors:** N. Gerasimova, D. La Civita, L. Samoylova, M. Vannoni, R. Villanueva, D. Hickin, R. Carley, R. Gort, B. E. Van Kuiken, P. Miedema, L. Le Guyarder, L. Mercadier, G. Mercurio, J. Schlappa, M. Teichman, A. Yaroslavtsev, H. Sinn, A. Scherz

**Affiliations:** a European XFEL, Holzkoppel 4, 22869 Schenefeld, Germany; ESRF – The European Synchrotron, France

**Keywords:** soft X-ray, monochromator, diffraction grating, FEL, beamline

## Abstract

The SASE3 soft X-ray beamline at the European XFEL has been designed and built to provide experiments with a pink or monochromatic beam in the photon energy range 250–3000 eV. Here, the focus is monochromatic operation of the SASE3 beamline, and the design and performance of the SASE3 grating monochromator are reported. The unique capability of a free-electron laser source to produce short femtosecond pulses of a high degree of coherence challenges the monochromator design by demanding control of both photon energy and temporal resolution. The aim to transport close to transform-limited pulses poses very high demands on the optics quality, in particular on the grating. The current realization of the SASE3 monochromator is discussed in comparison with optimal design performance. At present, the monochromator operates with two gratings: the low-resolution grating is optimized for time-resolved experiments and allows for moderate resolving power of about 2000–5000 along with pulse stretching of a few to a few tens of femtoseconds RMS, and the high-resolution grating reaches a resolving power of 10 000 at the cost of larger pulse stretching.

## Abstract 2

The SASE3 soft X-ray beamline at the European XFEL is equipped with a grating monochromator. The design and current performance of the monochromator are discussed.

## 1. Introduction

The SASE3 beamline at the European XFEL facility operates in the soft X-ray range from 250 eV to 3 keV (Altarelli *et al.*, 2006 ▸; Tschentscher *et al.*, 2017 ▸). The undulators of the SASE3 beamline produce femtosecond pulses of intense, highly coherent radiation that are transported to diverse experiments performed either at the SQS (Small Quantum Systems) instrument or at the SCS (Spectroscopy and Coherent Scattering) instrument; a third soft X-ray instrument SXP (Soft X-ray Port) is currently under construction. A considerable number of experiments conducted at the SASE3 beamline, in particular those applying spectroscopic techniques, demand for photon energy resolution substantially better than the typical 0.2–1% bandwidth of SASE (self-amplified spontaneous emission) radiation. In order to reduce the photon energy bandwidth at the experiments, the SASE3 beamline is equipped with a grating monochromator. The SASE3 beamline, along with the monochromator, has provided beam for experiments since the end of 2018. Here, we present the design of the SASE3 monochromator, its status as of 2021 and the performance achieved in the first three years of operation.

The design of a beamline at a free-electron laser (FEL) source is driven by the aim not to compromise the unique properties of the FEL pulses by the beam transport. The high degree of coherence of the FEL pulses entails a goal to enable close to diffraction-limited beam transport. This goal sets tight demands on the quality of optical elements. Furthermore, the European XFEL produces trains of pulses of millijoule intensity level at a high intra-train repetition rate of up to 4.5 MHz so that care should be taken to avoid damage to the optics. To reduce the risk of damage, particularly critical in the soft X-ray range, where the interaction of radiation with matter results in very high densities of excitations, it is expedient to increase the beam footprint on optics and use low-*Z* materials for coating of reflecting optical elements in order to reduce the absorbed dose. To allow large footprints of 4σ of a Gaussian-like beam profile, very long beamlines with very long optical elements are envisaged. Another property of FEL radiation is the ultrashort pulse duration of a few to a few tens of femtoseconds, which is important for a wide range of time-resolved experiments. The goal to minimize deterioration of temporal resolution by the monochromator sets additional demands on the grating of the monochromator. In contrast to a synchrotron beamline, where the monochromator design aims for the best compromise between photon energy resolution and transmission, in the case of an FEL source, pulse stretching by the grating should be taken into account as well. Since many spectroscopic experiments demand the best compromise between temporal and photon energy resolution, another aim is to minimize the time–bandwidth product of transported pulses. In order to minimize the time–bandwidth product and to transport close to transform-limited pulses, aberrations by optics should be minimized ideally down to the instrument response function of the ideal grating. This leads to very tight demands on the grating quality.

The design of the SASE3 monochromator is based on the combination of an elliptical mirror and a VLS (variable line spacing) grating. Reducing the number of optical elements involved to two minimizes the effect of the figure error of optics on performance. In order to minimize the pulse stretching by the grating, a line density of 50 lines mm^−1^ (l mm^−1^) was chosen as the lowest possible to be produced by the start of operation of the European XFEL facility. The design foresees achieving >4σ of Gaussian-like beam profile transmission in the substantial part of the photon energy range of SASE3 operation. However, achieving the design parameters for a 500 mm-long VLS grating was outside of production capabilities at the time. Therefore, a 120 mm-long 50 l mm^−1^ grating, allowing for moderate photon energy resolution along with minimal pulse stretching, was produced and installed by the start of operation. In 2021, the beamline was complemented by a 120 mm-long 150 l mm^−1^ grating, allowing for higher photon energy resolution at a cost of larger pulse stretching. The performance of the two gratings in operation is discussed in the present work in comparison with the optimal design performance.

## 2. Layout of the SASE3 monochromator

**Figure 1:**
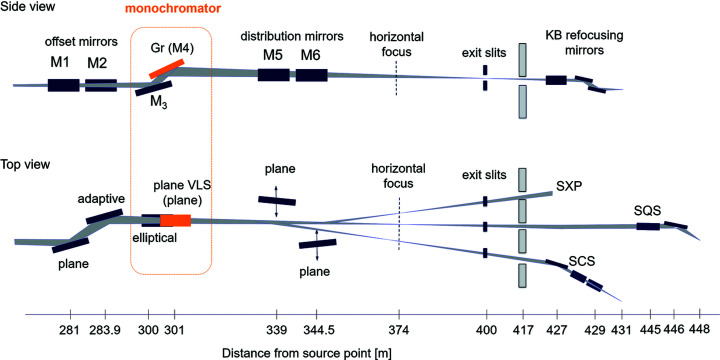
Layout of the SASE3 beamline, adapted from the work by Sinn *et al.* (2012 ▸).

The concept of the SASE3 monochromator was introduced by Sinn *et al.* (2012 ▸). The layout of the SASE3 beamline is presented in Fig. 1 ▸; details on the optical elements are summarized in Tables 1 ▸ and 2 ▸. The offset mirrors (M1 and M2) and the distribution mirrors (M5 and M6) deflect the beam in the horizontal plane and serve for the beam transport to the instruments: switching from the SQS instrument to the SCS instrument is done by inserting mirror M5, and from the SQS instrument to the SXP instrument by inserting mirror M6. The adaptive mirror M2 allows for horizontal focusing of the beam. The SASE3 monochromator itself operates in the vertical plane and consists of two optical elements: a meridianally focusing premirror M3 and a plane VLS grating. The premirror of elliptic cylinder shape focuses the source point, located at the exit of the undulator system, onto the exit slit; the VLS ruling of the grating compensates for aberrations arising from the operation of the grating in the convergent beam. The monochromator operates at a fixed included angle; the two premirrors: the LE (low-energy) premirror, operating at a grazing angle of 20 mrad, and the HE (high-energy) premirror, operating at a grazing angle of 9 mrad, cover the photon energy range 250–3000 eV. Changing the photon energy is achieved by rotation of the grating. The internal diffraction orders are used. Finally, there is a possibility to use the plane mirror M4 instead of a grating so that a pink beam with the same focusing properties as the monochromatic beam is transported to the experiments.

All the optical elements of the SASE3 beamline are made of single-crystal silicon and coated with a 50–65 nm layer of B_4_C. The roughness of the substrates was specified as <0.2 nm root mean square (RMS), the tangential slope error of the mirrors as <50 nrad RMS and the height error of the mirrors as <1.5 nm for the vertically/horizontally deflecting optics. In order to reduce thermal deformations, the optical elements were designed to be cooled with an InGa eutectic bath (Sinn *et al.*, 2012 ▸; La Civita *et al.*, 2014 ▸). Up to now, the cooling has been implemented only for the horizontally deflecting mirrors.

The key element of the monochromator is the grating. The design of the monochromator, based on two optical elements only, minimizes the number of optical elements involved and thus reduces wavefront deformation due to figure error introduced by every optical element. On the other hand, such a design demands very high accuracy of the VLS parameters. In spite of existing advanced technologies of VLS grating production, there are two limiting particularities in the case of SASE3 grating. Firstly, to allow transmission >4σ of a Gaussian-like beam, the length of the grating was specified to be 500 mm, which is unusually long for VLS gratings. Importantly, the required accuracy of the VLS parameters increases drastically with the length of the grating. Secondly, in order to prevent damage of the optical elements and exit slits, the design of the beamline foresees very long distances: the distance from the nominal source to the grating is 301 m, the distance from the grating to the exit slit is 99 m. Such long focal distances result in very low VLS parameters. The low VLS parameters should not challenge grating production based on mechanical ruling; however, the setups for holographic grating production are oriented for larger values of VLS parameters. As a result, the designed 500 mm-long 50 l mm^−1^ grating happened to be beyond the production capabilities to be ready by the start of operation. As a working solution, a shorter 120 mm-long 50 l mm^−1^ grating was produced holographically by Horiba Jobin-Yvon. To allow for higher photon energy resolution, the beamline has been complemented recently by the 120 mm-long 150 l mm^−1^ grating. The characteristics of these gratings are listed in Table 2 ▸. Below we discuss the expected performance of the designed grating and compare it with the performance of the two gratings in operation.

Experiments with monochromatic beams can be performed either by selecting a fixed photon energy or by scanning the photon energy. During scanning, the motions of the monochromator and the SASE3 undulators are synchronized. In addition, the monochromator is regularly utilized as a spectrometer for spectral diagnostics of the FEL beam. The spectrometer operation mode is realized by introducing a YAG:Ce crystal into the focal plane of the monochromator. The YAG:Ce crystal converts X-ray photons into optical luminescence, which is registered in turn by a CCD coupled with a multi-channel plate (MCP) (Koch *et al.*, 2019 ▸). The resulting 2D images represent the spectral distributions in the focal plane of the monochromator.

## 3. Methods

The performance of the SASE3 monochromator has been evaluated analytically over the SASE3 operational range. To estimate the beam footprint on the optics, and the consequent geometrical cut in the vertical direction due to the limited grating length, the FEL beam was approximated by a Gaussian-like beam; the beam divergence was estimated according to the work by Sinn *et al.* (2011 ▸), based on the expected SASE beam properties in the case of the low electron beam charge of 20 pC presented by Schneidmiller & Yurkov (2011 ▸). The resulting shape of the footprint on the grating [cut Gaussian-like beam – see inserts in Fig. 4(*a*)] was used to estimate the pulse stretching: *t* = (*M* *k*λ*F*)/*c*, where *M* is the diffraction order, *k* is the line density, λ is the photon wavelength, *c* is the speed of light and *F* is the beam footprint on the grating. The corresponding beam profile after the grating was used to retrieve the monochromatic instrument response function (IRF) of an ideal grating in the exit slit plane by Fourier transform. Knowing the focal distance, angular distributions obtained by Fourier transform were converted to spatial dimensions. To obtain the IRF of a real grating, the IRF of an ideal grating should be convoluted with a function representing aberrations. Aberrations due to slope error on optics and due to miscut of the VLS parameters were taken into account. A simplified formula, assuming Gaussian shapes of the IRF and of the aberrations, was used to estimate the FWHM (full width at half-maximum) of the IRF of the real grating: *irf* = (*irf*_gr_^2^ + *a*_slope_^2^ + *a*_vls_^2^)^1/2^, where *irf*_gr_ is the FWHM of the IRF of an ideal grating given by the beam footprint on the grating, *a*_slope_ is the aberration induced by the slope error, and *a*_vls_ is the aberration induced by the VLS miscut. The aberration induced by the slope error was estimated as a combination of the aberration due to slope error on the premirror *a*_slope_pm_ = 2σ_slope_pm_/*cff* and the aberration due to slope error on the grating *a*_slope_gr_ = σ_slope_gr_(1 + *cff*)/*cff*, where *σ*_slope_pm_ and *σ*_slope_gr_ are the slope errors on the premirror and on the grating, respectively, *cff* = cosβ/cosα is the fixed focus constant, α is the angle of incidence on the grating, and β is the angle of diffraction. The resolution relates to the FWHM of IRF: *r* = *irf* *dE*, where *dE* is the dispersion. The target VLS parameters and aberrations induced by miscut of the VLS parameters have been estimated analytically using the geometrical theory of diffraction grating (Noda *et al.*, 1974 ▸). To estimate tolerances on the VLS parameters, aberrations induced by each VLS term have been set equal to half of the FWHM of the IRF of the grating with ideal VLS. Such a condition would result in a resolution reduced by ∼11% in the case of a Gaussian profile. The details can be found in the study by Gerasimova (2018 ▸) on the 50 l mm^−1^ grating.

To confirm analytical estimations of IRF and resolution, wavefront propagation simulations have been applied for several working points. The WaveProperGator (WPG) framework (Samoylova *et al.*, 2016 ▸) was used; the source was modelled as a Gaussian-like beam. When departing from optimal conditions (*e.g.* due to imperfections of the grating or defocus), the shape of the IRF becomes ambiguous, so the reduction of main peak intensity was used as a quantitative measure of resolution deterioration. The wavefront propagation simulations were used for the investigation of the longitudinal focusing properties, such as the dependence of resolution on the premirror focusing distance in the case of a grating with residual curvature.

For estimating the time–bandwidth product, which is a product of the FWHM pulse duration and the FWHM photon energy bandwidth after the monochromator, the pulse duration after the monochromator was assumed to be equal to the pulse stretching by the grating, and the FEL pulse duration itself was neglected. This approximation is valid for the cases in which the pulse stretching is longer than the FEL pulse duration at a fixed photon energy; the latter is typically on the order of 10 fs FWHM and below, as estimated by the spectral correlations technique.

The transmission of the beamline, representing the ratio of the intensity on the sample to the total FEL intensity, has been estimated by combining (i) the geometrical cut by the optics, (ii) the grating efficiency and mirrors reflectivity, and (iii) the spectral transmission through the exit slit. Grating efficiencies have been calculated using *Reflec* code (Schäfers & Krumrey, 1996 ▸), which is part of the *Ray* package (Schäfers, 1996 ▸) developed at the Berliner Elektronenspeicherring-Gesellschaft für Synchrotronstrahlung (BESSY). To estimate the spectral transmission through the exit slit, the exit slit was assumed to be centred on the maximum of the Gaussian-like average spectrum of 1% bandwidth. In practice, the average FEL spectra differ from Gaussian shape and could have various bandwidths (from 0.2–0.5% of the theoretical SASE bandwidth to >1%). To compare measurements and estimations, the SASE average spectrum was measured in every experimental point, and the spectral transmission through the exit slit of the measured spectrum was normalized to the spectral transmission of the Gaussian-like spectrum of 1% bandwidth. The transmission was measured as the ratio of average intensities, registered by two X-ray gas monitor (XGM) detectors (Maltezopoulos *et al.*, 2019 ▸) located before the first beamline mirror M1 and after the SCS exit slit.

Absorption resonances at the Ne *K*-edge were used for monochromator calibration and for photon energy resolution measurements. The measurements were made in transmission: the 15 m-long gas attenuator (Villanueva *et al.*, 2022 ▸) located upstream of the monochromator was filled with Ne gas to a pressure of 0.005–0.03 mbar, depending on the monochromator configuration. The beamline was operating in spectrometer mode so that single-shot spectra were acquired in the focal plane of the monochromator by a CCD gated with an MCP. The spectra were taken at 10 Hz, an ensemble of 5000–10000 single-shot spectral distributions were averaged, and the resulting spectrum was normalized by an average FEL spectrum. The resulting transmission spectra were fit with a function resulting from a convolution of the modelled Ne *K*-edge transmission spectrum with the Gaussian line representing the IRF of the monochromator. To model the transmission in the 1*s* excitation region of Ne, the absorption cross-section was modelled according to De Fanis *et al.* (2002 ▸) by Lorentzian lines of natural lifetime width Γ = 240 meV, located at 867.12 eV (1*s* ^−1^3*p*), 868.69 eV (1*s* ^−1^4*p*), 869.27 eV (1*s* ^−1^5*p*) *etc*. The photon energy resolution was estimated as an FWHM of the IRF of the monochromator resulting from the fit.

## 4. Performance of the SASE3 monochromator

### 4.1. Photon energy resolution and temporal resolution: design and limitations

The photon energy resolution was estimated analytically over the SASE3 operation photon energy range, as described in Section 3. To confirm the analytical estimations, wavefront propagation simulations were performed at several working points.

**Figure 2:**
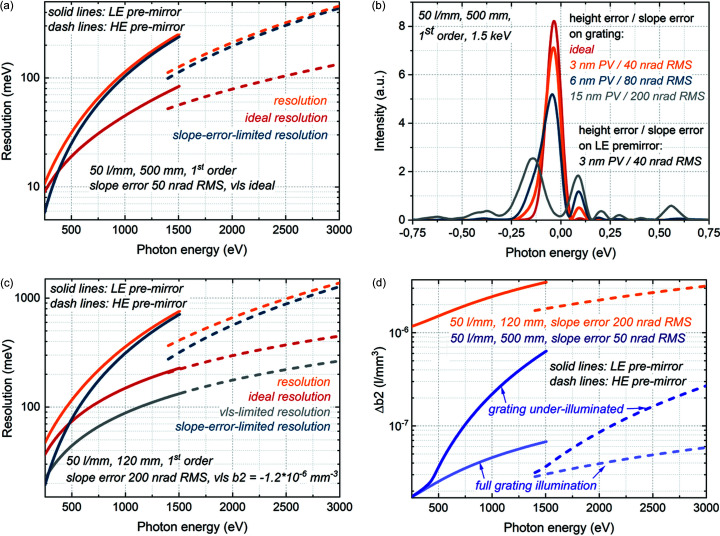
Influence of figure error and VLS miscut on resolution. (*a*, *c*) Analytically estimated resolution of the monochromator operating in first diffraction order with designed and installed 50 l mm^−1^ gratings; slope error on premirror 50 nrad RMS. (*b*) Instrument response function of the 500 mm-long 50 l mm^−1^ designed grating operating in first diffraction order at 1.5 keV obtained by wavefront propagation simulations for the cases of different magnitudes of slope error on the grating. (*d*) Analytically estimated tolerances on the VLS *b*2 parameter for the 500 mm-long 50 l mm^−1^ designed grating and for the 120 mm-long 50 l mm^−1^ grating.

The influence of the figure error on photon energy resolution in the case of the 500 mm-long 50 l mm^−1^ designed grating operating in first diffraction order is presented in Figs. 2 ▸(*a*) and 2(*b*). The 50 nrad RMS slope error was specified for the premirrors and for the designed grating; this value is currently at the limit of production and metrology capabilities. Analytical estimations show that a combination of the aberrations due to 50 nrad slope error on the premirror and the aberrations due to 50 nrad slope error on the grating results in the slope-error-limited resolution dominating over the resolution of the ideal grating [Fig. 2 ▸(*a*)]. The high photon energy range is more strongly influenced by the slope error, so that a deterioration of the overall resolution due to slope error is more substantial at higher photon energy ranges of operation for each premirror. The influence of the magnitude of the figure error on the resolution of the 500 mm-long 50 l mm^−1^ grating was also investigated by wavefront propagation simulations. In contrast to the analytical estimations, where the slope error introduces an angular spread of deflected rays with a consequent focus deterioration, in the wavefront propagation simulations the height error is introduced as a phase screen resulting in wavefront distortion of coherent beam and consequent focus deterioration. The results of wavefront propagation show lower influence of figure error compared with analytical estimations: as it can be seen in Fig. 2 ▸(*b*), in the case of 1500 eV, the 3 nm height error corresponding to the 40 nrad slope error on the grating would only lead to a small reduction of resolution compared with an ideal slope on the grating.

The estimations of resolution of the 120 mm-long 50 l mm^−1^ grating operational in the first diffraction order are presented in Fig. 2 ▸(*c*). Due to the shorter grating length compared with the 500 mm-long designed grating, fewer grooves are illuminated, resulting in broader IRF and lower ideal resolution. Consequently, the worse slope error of 200 nrad RMS leads to a similar relative deterioration of resolution in the case of a 120 mm-long grating as a 50 nrad RMS slope error in the case of a 500 mm-long grating. The influence of VLS miscut on the resolution was studied as well. The optimal VLS *b*2 for the 50 l mm^−1^ grating is 1.53 × 10^−8^ l mm^−3^; the lowest VLS *b*2 achieved in the production of a 120 mm-long 50 l mm^−1^ grating is −1.2 × 10^−6^ l mm^−3^. The aberrations induced by this miscut are inferior to the ideal resolution and do not limit the resolution of the 120 mm-long 50 l mm^−1^ grating operational in first diffraction order [Fig. 2 ▸(*c*)]. However, in second diffraction order this VLS *b*2 miscut is already a limiting factor for the resolution of this grating.

Let us discuss the VLS parameters, which limit the production of the designed grating. The tolerances on the VLS parameters were estimated as described in Section 3 by setting the VLS-induced aberration equal to half the FWHM of the IRF of the grating with ideal VLS. The resulting tolerances on the VLS *b*2 parameter are presented in Fig. 2 ▸(*d*). Important to underline here is the strong dependence of the illuminated grating length on the magnitude of aberrations induced by the VLS miscut: the aberrations due to a miscut of *b*1 would be proportional to the first power of the illuminated grating length, *b*2 to the second power of the grating length, and *b3* to the third power of the grating length *etc*. In addition, the FWHM of the IRF of an ideal grating is inversely proportional to the grating length, demanding smaller aberrations for longer gratings. Therefore, the tolerance to be set on the VLS *b*2 parameter would be approximately proportional to the third power of the illuminated grating length. As shown in Fig. 2 ▸(*d*), if the full grating is illuminated, the tolerances on the VLS *b*2 for a 500 mm-long grating are almost two orders of magnitude tighter than the tolerances for a 120 mm-long grating. However, due to the photon-energy-dependent FEL beam divergence and the monochromator geometry, the 500 mm-long grating is under-illuminated at higher photon energy ranges of operation for each premirror [Fig. 4(*a*)]. When only partial illumination of the grating, corresponding to the FWHM of the beam footprint, is taken into account to estimate aberrations due to the VLS parameters, the tolerances in the case of the 500 mm-long grating become less tight.

**Figure 3:**
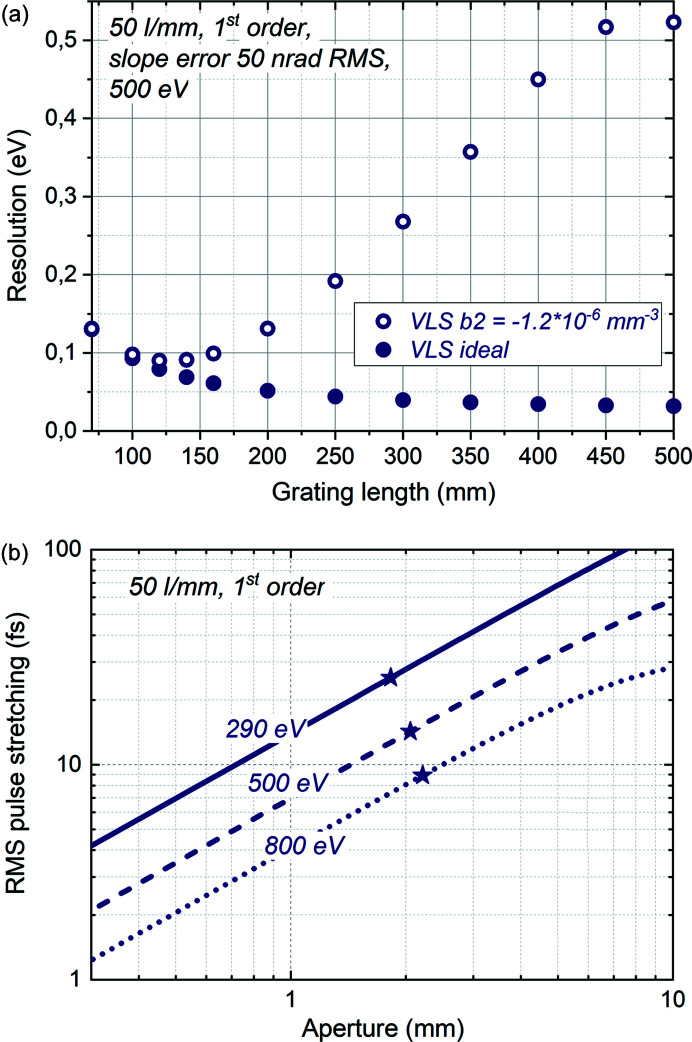
Influence of grating length on resolution and pulse stretching. (*a*) Dependence of analytically estimated resolution of the 50 l mm^−1^ grating with 50 nrad RMS slope error, operating in first diffraction order at 500 eV on the grating length. Solid circles – ideal VLS; open circles – VLS *b*2 = −1.2 × 10^−6^ l mm^−3^. (*b*) Pulse stretching versus open aperture upstream of the 50 l mm^−1^ grating operating in first diffraction order: solid line – 290 eV; dashed line – 500 eV; dotted line – 800 eV. The stars correspond to 120 mm length of grating illumination.

The strong dependence of aberrations induced by a miscut of the VLS parameters on the grating length discussed above is important to understand the limitations set by grating production capabilities on reasonable grating length. While increasing the length and the illuminated part of an ideal grating will improve the resolution, the slope error would stop this improvement when the resolution becomes slope-error-limited. The effect of the VLS miscut on resolution is even stronger and would lead to deterioration of the resolution with increase in the grating length as soon as the resolution becomes VLS-miscut-limited. This tendency can be seen in Fig. 3 ▸(*a*), where the analytically estimated resolution of the 50 l mm^−1^ grating with 50 nrad slope error operating in first diffraction order is presented for the cases of ideal VLS and VLS *b*2 = −1.2 × 10^−6^ l mm^−3^. In the latter case, the best resolution would be achieved at a grating length of about 130 mm and would deteriorate with further increase in the grating length, *e.g.* at 400 mm grating length, the resolution would be spoiled by a factor of five compared with optimal resolution at 130 mm length. For the 150 l mm^−1^ 120 mm-long grating, produced a few years later after the 50 l mm^−1^ 120 mm-long grating, tighter VLS *b*2 = −8.7 × 10^−8^ l mm^−3^ has been achieved. Such a small VLS miscut does not introduce any impact on resolution for the operation in first diffraction order, and, assuming a longer grating with such VLS parameters could be produced, it would be possible to increase the grating length to 250 mm without resolution deterioration.

Another effect accompanying an increase in the grating length is an increase in the pulse stretching. The grating introduces pulse stretching due to the pulse front tilt resulting from the difference between the angle of incidence and the angle of diffraction. Since this difference becomes more pronounced at lower photon energies, pulse stretching would be more pronounced at lower photon energies. For a given line density, pulse stretching depends on the size of the illuminated part of the grating: the longer the illuminated part of the grating, the longer the pulse stretching. The illuminated part of the grating could be controlled by an aperture introduced before the grating. The dependence of pulse stretching by a 50 l mm^−1^ grating on the aperture size is presented in Fig. 3 ▸(*b*) for operation at 290 eV, 500 eV and 800 eV. The stars correspond to the 120 mm length of the grating. Importantly, while the pulse stretching is given by geometry only and does not depend on the quality of the grating, the resolution degrades compared with best possible resolution with an increase in the grating length. This results in an increase of the time–bandwidth product with the increase of the length of a real grating, starting from the point at which aberrations begin to influence the resolution.

**Figure 4:**
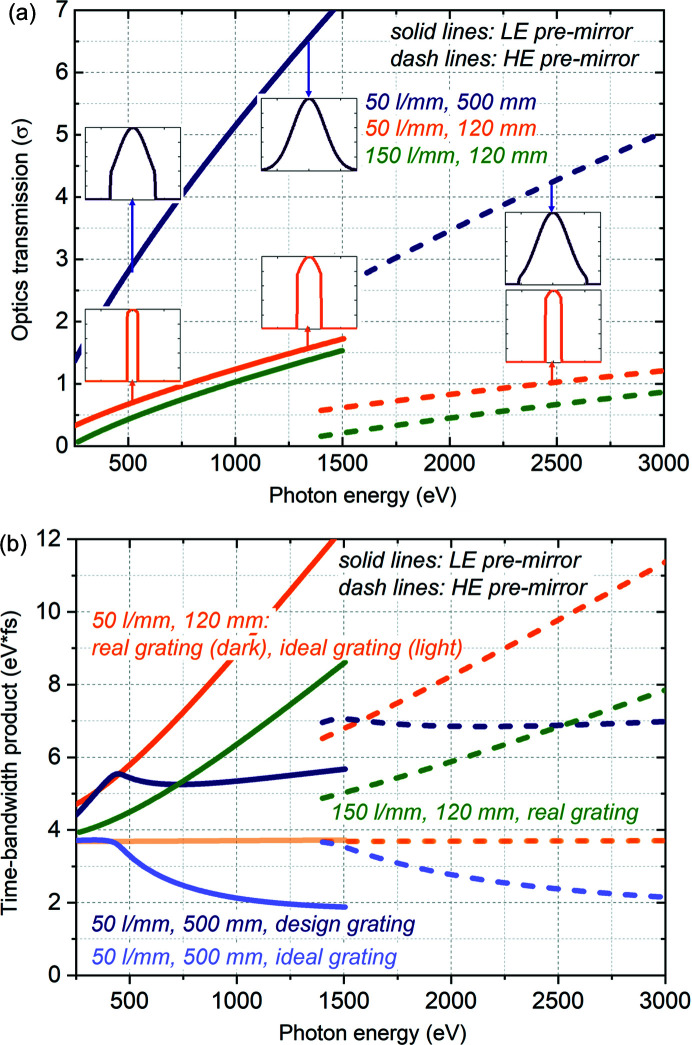
Time–bandwidth product. (*a*) Analytically estimated optics transmission in sigma of Gaussian-like beam cross-section; the inserts show the shape of the beam transmitted by the monochromator. (*b*) Analytically estimated time–bandwidth product in the case of ideal optics with a perfect slope and a perfect VLS law, as well as in the case of the designed grating and installed gratings. Operation in first diffraction order.

Let us discuss the time–bandwidth product in detail. As pointed out above, one of the design goals was to minimize the time–bandwidth product in order to provide experiments with the best compromise between temporal and photon energy resolution. First of all, in the case of ideal optics, the time–bandwidth product depends on the shape of the beam footprint on the grating only. It is well known that the time–bandwidth product is minimal for a Gaussian-like beam, reaching ∼1.83 eV fs, whereas it becomes almost twice as large for a flattop-like beam, resulting in a sinc-like IRF. Therefore the optimal performance could be achieved for a transmission of >4σ of a Gaussian-like beam cross-section. The estimated geometrical transmission of the beamline in the vertical direction, given by the grating open aperture and the beam divergence, is shown in Fig. 4 ▸(*a*) for the cases of 500 mm-long and 120 mm-long gratings; the corresponding shape of the transmitted cut Gaussian-like beam is shown in inserts at a few points. Operation of the monochromator at constant included angle results unavoidably in a decrease in geometrical transmission at the low photon energy side of the operation range; moreover, a substantial increase in divergence towards low photon energies decreases transmission at low photon energies further. One can see that a 500 mm-long grating would allow transmission of >4σ of the Gaussian-like beam cross-section for a substantial part of the operation range. In contrast, with the currently operational 120 mm-long grating, the transmission is below 1.7σ over the full operation range. A decreased transmission not only decreases flux on the sample but also results in a less optimal time–bandwidth product, as can be seen in Fig. 4 ▸(*b*), where the estimated time–bandwidth product is presented for the cases of ideal optics and design or real optics. For real gratings, the time–bandwidth product increases compared with that for ideal gratings, since the resolution would degrade due to aberrations while the pulse stretching would stay the same. That is why the ultimate goal would be to keep all manner of aberrations below the IRF of the ideal grating. In the case of the installed gratings of the SASE3 monochromator, the time–bandwidth product increases towards higher photon energy ranges of operation of each premirror as a consequence of increasing influence of slope error. The time–bandwidth product is smaller in the case of the 150 l mm^−1^ grating due to its lower slope error and lower VLS miscut. Limiting the grating illumination by closing the aperture before the grating in order to reduce pulse stretching would improve the time–bandwidth product. Finally, to characterize the time–bandwidth product at the experiment, the measured resolution should be taken into account instead of the calculated one.

### 4.2. Photon energy resolution and temporal resolution: performance

**Figure 5:**
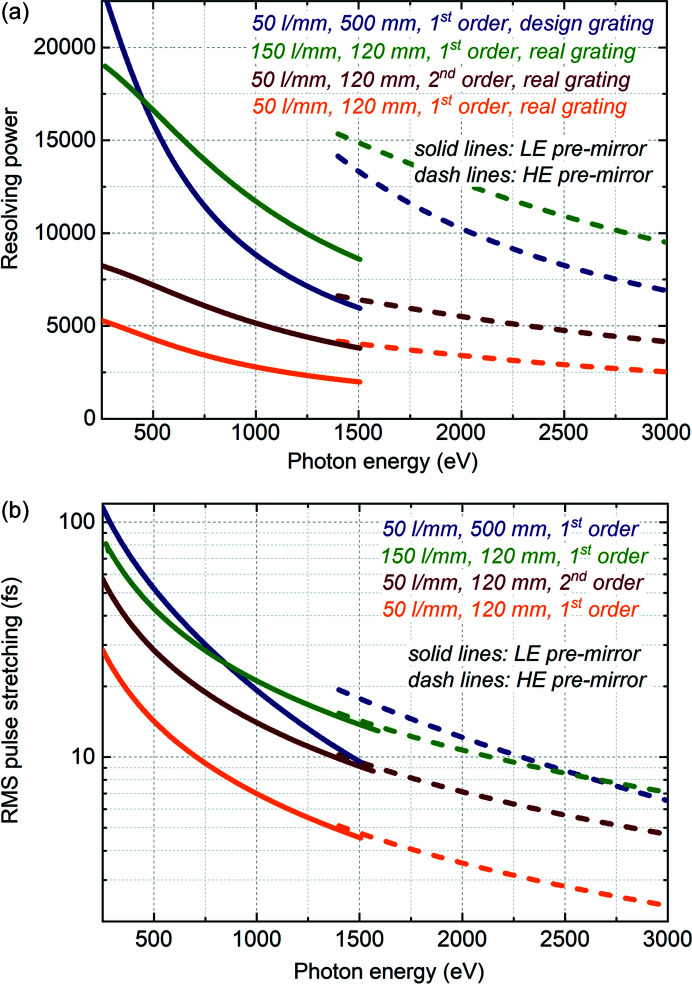
Monochromator performance. (*a*) Analytically estimated resolving power and (*b*) pulse stretching due to the grating for the 50 l mm^−1^ 500 mm-long designed grating operating in first diffraction order, for the 50 l mm^−1^ 120 mm-long installed grating operating in first and second diffraction orders, and for the 150 l mm^−1^ 120 mm-long installed grating operating in first diffraction order.

Estimations of resolving power and pulse stretching for the 50 l mm^−1^ and 150 l mm^−1^ 120 mm-long installed gratings in comparison with the 50 l mm^−1^ 500 mm-long designed grating are presented in Fig. 5 ▸. In addition to operation in first diffraction order, operation in second diffraction order is considered for the case of the 50 l mm^−1^ 120 mm-long grating because of the regular usage of this mode for experiments demanding strong suppression of higher FEL harmonics. The estimations were carried out by taking into account the slope error and VLS miscut affecting the resolution, as described in the previous subsection. The 50 l mm^−1^ 500 mm-long designed grating was meant to achieve about 10000 resolving power. Fig. 5 ▸ shows that such a resolving power about 10 000 should be achievable with the 150 l mm^−1^ 120 mm-long installed grating, although accompanied with lower transmission compared with the designed grating. The 150 l mm^−1^ grating is optimized for experiments demanding high photon energy resolution rather than high temporal resolution. On the other hand, the 50 l mm^−1^ 120 mm-long grating operating in first diffraction order should allow for moderate resolving power of 2000–5000 along with smaller pulse stretching in the range of a few to a few tens of femtoseconds RMS. This grating is optimized for experiments demanding high temporal resolution. Although, in the low photon energy range, the pulse stretching by this grating is still quite noticeable compared with typical FEL pulse duration. For experiments demanding shorter pulses, there is a possibility to reduce the pulse stretching by closing the aperture located prior to the grating, as shown in Fig. 3 ▸(*b*). Finally, operation of the 50 l mm^−1^ 120 mm-long grating in second diffraction order allows us to increase the resolving power compared with operation in first diffraction order, although this increase is limited by the VLS miscut.

**Figure 6:**
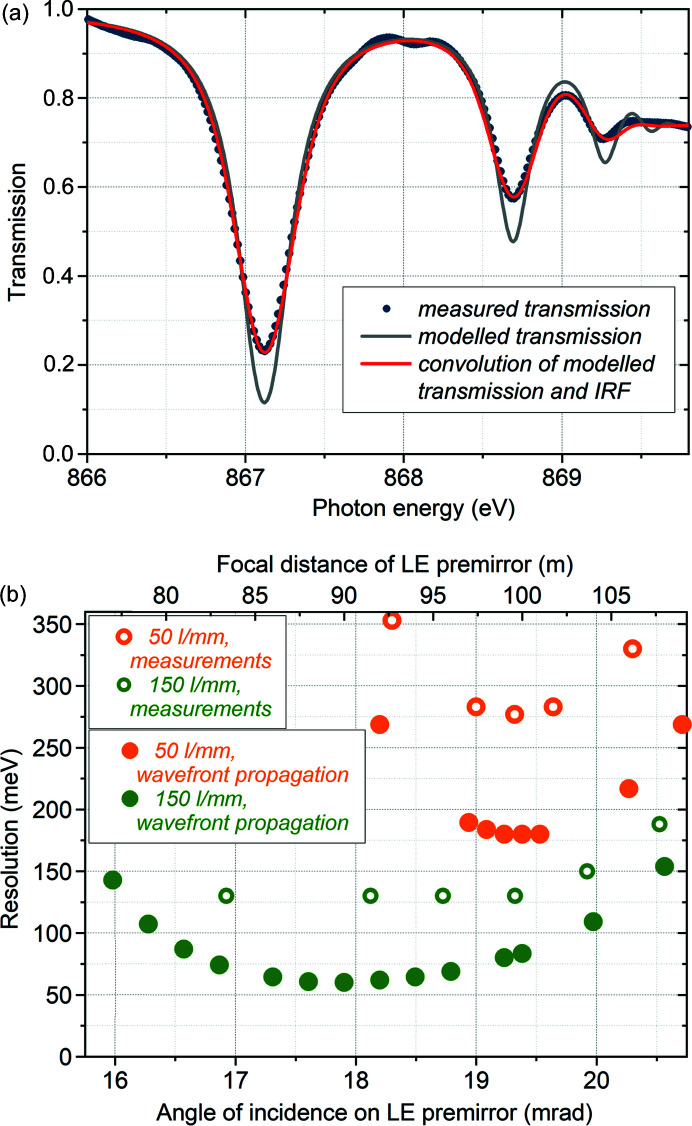
Resolution measurements and longitudinal focusing. (*a*) Transmission spectrum of Ne around the *K*-edge; circles represent measured transmission spectra; grey lines – modelled transmission; red lines – resulting convolution of modelled transmission with the monochromator IRF. (*b*) Resolution optimization at the Ne *K*-edge by aligning the angle of incidence of the LE premirror; operation in first diffraction order.

The photon energy resolution of the two installed gratings was evaluated and optimized using absorption resonances at the Ne *K*-edge, as described in Section 3. A typical transmission spectrum at the Ne *K*-edge is shown in Fig. 6 ▸(*a*). To optimize the resolution, the longitudinal focusing by the LE premirror has been optimized by changing its angle of incidence. The results of this optimization are shown in Fig. 6 ▸(*b*), where the open circles represent the resolution, estimated from measurements as described above, versus angle of incidence on the LE premirror. The upper scale shows the longitudinal focus position of the premirror, corresponding to the angle of incidence. The solid circles represent the results of wavefront propagation simulations. In the case of the 50 l mm^−1^ grating, the grating was assumed to be plane (the radius of curvature is equal to infinity), and the optimal focusing distance of the premirror corresponds to the focal distance of the monochromator. However, the radius of curvature of −198 km of the 150 l mm^−1^ grating (Table 2 ▸) shifts the focus downstream, therefore the premirror should be aligned in a way to compensate for this shift. The results of wavefront propagation show that, around 867 eV, the focusing of the LE premirror should be moved upstream by ∼10 m, achievable by decreasing the angle of incidence by ∼1.4 mrad. Similar results have been obtained analytically. Furthermore, the influence of residual curvature of the grating on the longitudinal focusing is photon-energy-dependent; the compensation by the premirror angle becomes more difficult at low photon energies. Thus, at 500 eV one has to move the focusing by the premirror upstream by an additional several meters, which becomes difficult to achieve by adjusting the angle of the premirror because of the geometrical constraints of the monochromator. The dependence of the measured resolution on the angle of incidence of the LE premirror is very similar to those estimated by wavefront propagation. The measurements in the case of the 150 l mm^−1^ grating are limited by the natural bandwidth of Ne lines; the minimal resolution that could be measured with this method was 130 meV, therefore the optimal angle of incidence on the LE premirror was chosen based on the wavefront propagation simulations for this grating.

The optimized resolving power around 867 eV, measured as described above, is ∼3300 for the 50 l mm^−1^ grating operating in first diffraction order and ∼4900 for the 50 l mm^−1^ grating operating in second diffraction order. For the 150 l mm^−1^ grating, the measured resolving power of 6700 is limited by the measurement technique. Yet, the resolving power of the 150 l mm^−1^ grating operating in first diffraction order has been proven to be >10000 in the range 500–950 eV. This was done by measuring the combined resolution with the hRIXS spectrometer; these measurements showed a resolving power of ∼10000 at both the O *K*-edge (∼530 eV) and the Cu *L*-edge (∼930 eV) (Schlappa *et al.*, 2022 ▸). For both gratings, the measured resolving power is in a good agreement with analytical estimations [presented in Fig. 5 ▸(*a*)].

### 4.3. Transmission

**Figure 7:**
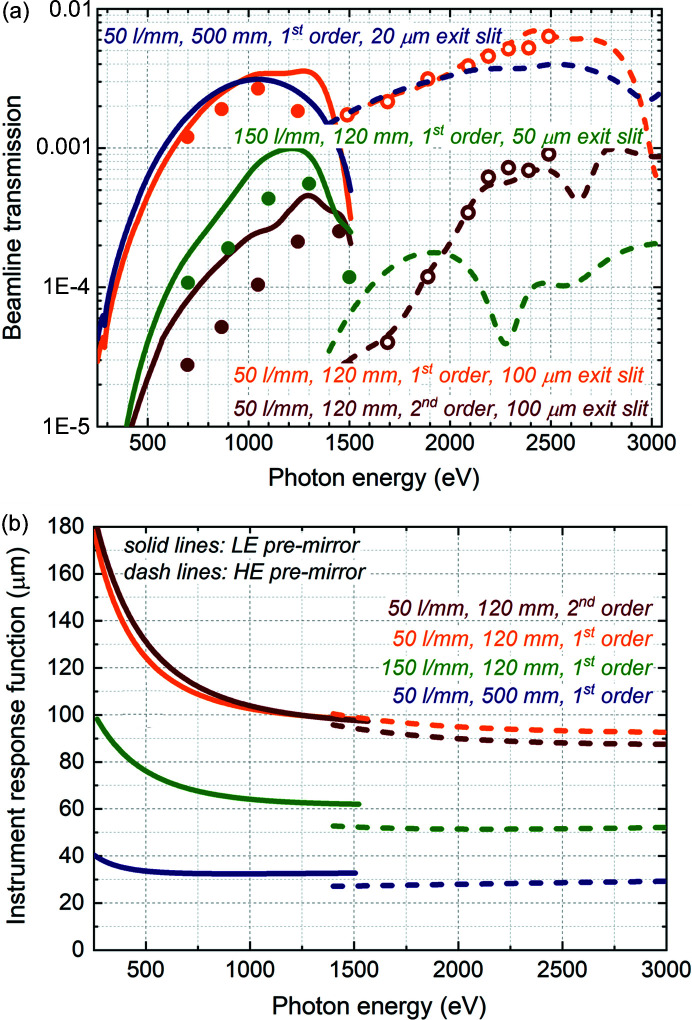
Transmission. (*a*) Estimated (lines) and experimentally measured (circles) beamline transmission operating with the 50 l mm^−1^ 500 mm-long designed grating (20 µm exit slit), with the 50 l mm^−1^ 120 mm-long installed grating (100 µm exit slit) and with the 150 l mm^−1^ 120 mm-long installed grating (50 µm exit slit). Solid lines and solid circles – operation with LE premirror; dashed lines and open circles – operation with HE premirror. (*b*) Instrument response function of respective gratings.

The estimated and measured transmission of the beamline operating in monochromatic mode is shown in Fig. 7 ▸(*a*). The transmission is presented through the exit slits of different widths for different gratings; the slit width is chosen based on the IRF presented in Fig. 7 ▸(*b*): 20 µm for the 50 l mm^−1^ 500 mm-long designed grating, 50 µm for the 150 l mm^−1^ 120 mm-long installed grating and 100 µm for the 50 l mm^−1^ 120 mm-long installed grating. Note that, because of the larger IRF, the wider slit width can be used without deterioration of resolution at lower photon energies where the transmission is low. In Fig. 7 ▸(*b*), the IRF corresponds to the estimated resolution whereas, based on the resolution measurements presented above, a broadening by a factor of ∼1.5 IRF is expected in practice.

There is excellent agreement between the measurements of transmission and the estimations for the case of operation with the HE premirror. The measured transmission in the case of the LE premirror, performed a year later, is lower than the estimated one, which may be an indication of slow contamination of the beamline optics.

## 5. Towards optimal performance

As can be observed from the discussion above, provided a grating of ultimate quality can be produced, an increase of the grating length could improve not only transmission but also time–bandwidth product. In practice, approaching the design performance by implementing a longer grating is a very challenging task. First of all, one has to overcome limitations in the grating production. The VLS *b*2 parameter became critical for the holographic grating production. In the case of mechanical ruling, since the VLS *b*2 parameter can be set to 0 within tolerances, the target mean value should be easily achievable. However, mechanical ruling is currently much less developed, compared with holographic ruling, and it suffers from other problems, such as large statistical deviations from the mean values of the ruling parameters over the grating length. In addition, attention has to be paid to the residual curvature of the grating since it shifts the longitudinal focus from the focal plane. Compensation of such a shift by changing the angle of the premirror (discussed in Section 4.2) is possible only in a limited range. The influence of residual curvature on resolution would increase strongly with an increase in the grating length because of a strong decrease of depth of focus for longer grating, allowing for larger acceptance and tighter focusing at the same time. Similarly, all the other factors influencing the longitudinal focusing become much more pronounced and, at some point, critical, with increasing grating length. One such factor is the position of the source in the undulator system. If, hypothetically, the shift of focus could be corrected by introducing adaptive properties to the premirror, the prolongation of the source would unavoidably lead to resolution deterioration when approaching the ultimate performance. The longitudinal properties of the source could become critical even for smaller gratings sizes in certain XFEL operation modes, such as strong quadratic tapering. Further limitations could arise due to induced thermal deformations (bump) of the long grating surface when using the full power of the XFEL beam (La Civita *et al.*, 2014 ▸).

## 6. Summary

The SASE3 beamline is equipped with a grating monochromator allowing reduction of the photon energy bandwidth at the experiments and improvement of the limited longitudinal coherence of SASE radiation. The monochromator has been designed with the goal to minimize the time–bandwidth product and to transport close to transform-limited pulses. At present, the monochromator is equipped with two gratings: a low-resolution grating, optimized for time-resolved experiments and allowing for moderate resolving power of about 2000–5000 along with pulse stretching of a few to a few tens of femtoseconds RMS, and a high-resolution grating, reaching a resolving power of 10000 at a cost of larger pulse stretching.

## Figures and Tables

**Table 1:** Optical elements of the SASE3 beamline: mirrors

	Horizontally deflecting optics		Vertically deflecting optics	
	M1/M2, offset mirrors	M5/M6,† distribution mirrors	M3a/M3b, focusing premirrors	M4, deflection mirror
Optical surface size (mm)	850 × 20	850 × 20	580 × 25	500 × 30
Shape	Plane/adaptive	Plane	Elliptic cylinder	Plane
Tangential slope error, RMS (nrad)	56/58	75/50	63/37	140
Tangential height error, PV (nm)	1.6/1.9	1.9/2	2.1/2.6	7
Roughness, RMS (nm)	0.14/0.13	0.2/0.2	0.23/0.15	0.15
Grazing angle of incidence (mrad)	(9–20)	9	9/20	9, 20

† Specification.

**Table 2:** Optical elements of the SASE3 beamline: gratings

Grating	LR Gr, low-resolution designed grating†	LR Gr, low-resolution installed grating	HR Gr, high-resolution installed grating
Tangential radius of curvature (km)	–	>300	−198
Optical surface size (mm)	500 × 25	120 × 17	120 × 20
Slope error, RMS (nrad)	<50	200	130
Height error, PV (nm)	<3	6	3.6
Roughness, RMS (nm)	<0.2	<0.2	<0.6
Included angle (mrad)	π − 18, π − 40		
VLS law:	*n*(*w*) = *b*0 + *b*1*w* + *b*2*w* ^2^+…		
Central line density *b*0 (l mm^−1^)	50	50.05	150
*b*1 (l mm^−2^)	1.01 × 10^−3^	1.0 × 10^−3^	3.029 × 10^−3^
*b*2 (l mm^−3^)	0. l	−1.2 × 10^−6^	−8.7 × 10^−8^
Groove profile	Blazed, 0.1° blaze angle	Laminar, 16 nm depth	Laminar, 16 nm depth

† Specification.
